# Therapeutic Effects of Glucagon-like Peptide-1 Receptor Agonists in Non-Alcoholic Fatty Liver Disease: A Systematic Review

**DOI:** 10.3390/ijms27125618

**Published:** 2026-06-22

**Authors:** Dina Mahoon, Fares Kellany, Imad Khan, Somieya Khan, Alexandra E. Butler

**Affiliations:** 1 School of Medicine, Royal College of Surgeons in Ireland-Medical University of Bahrain, Busaiteen 15503, Bahrain; 22203142@rcsi.com; 2 Research Department, Royal College of Surgeons in Ireland-Medical University of Bahrain, Busaiteen 15503, Bahrain; 23200118@rcsi.com (F.K.); 23203692@rcsi.com (I.K.); 20205059@rcsi.com (S.K.)

**Keywords:** NAFLD, MASLD, GLP-1 receptor agonists, liver fat, liver fibrosis

## Abstract

Non-alcoholic fatty liver disease (NAFLD), now increasingly termed metabolic dysfunction-associated steatotic liver disease (MASLD), is a growing cause of chronic liver disease with limited treatment options. Glucagon-like peptide-1 (GLP-1) receptor agonists, approved for type 2 diabetes and obesity, possess metabolic effects that may render them suitable for treating NAFLD and metabolic dysfunction-associated steatohepatitis (MASH). To evaluate the therapeutic effects of GLP-1 receptor agonists in adults with NAFLD, non-alcoholic steatohepatitis (NASH), MASLD, or MASH. PubMed, Scopus, Embase, and the Cochrane Library were systematically searched using keywords related to NAFLD and GLP-1 receptor agonists. Given heterogeneity in populations, designs, and outcomes, findings were synthesized narratively. The review is registered with PROSPERO (CRD420261337353). Twelve studies met the inclusion criteria. The most consistent outcome was a reduction in hepatic fat, seen with semaglutide, liraglutide, dulaglutide, and beinaglutide. Improvements in liver enzymes, particularly alanine aminotransferase, were less consistent and best regarded as supportive rather than definitive evidence of histological improvement. Histological benefits were strongest for steatohepatitis resolution in non-cirrhotic MASH. Fibrosis findings were mixed, with the greatest benefit in F2–F3 MASH and limited improvement in established cirrhosis. GLP-1 receptor agonists were generally well tolerated, with gastrointestinal symptoms the most common adverse effects. GLP-1 receptor agonists show promising liver-related benefits in NAFLD and MASH, particularly in obesity, type 2 diabetes, or earlier-stage disease. Their effects on advanced fibrosis and long-term outcomes remain uncertain, warranting larger, longer-term studies.

## 1. Introduction

### 1.1. Non-Alcoholic Fatty Liver Disease

Non-alcoholic fatty liver disease (NAFLD) is the most prevalent chronic liver disease globally and constitutes a major public health concern [1,2]. It is defined by an excessive amount of hepatic fat accumulation in individuals without substantial alcohol intake or other secondary causes of hepatic steatosis [3]. NAFLD encompasses a spectrum of clinical and pathological conditions, ranging from isolated hepatic steatosis to non-alcoholic steatohepatitis (NASH), which involves hepatocellular injury, inflammation, and varying degrees of fibrosis [3]. Progressive fibrosis can ultimately lead to hepatic consequences such as cirrhosis, hepatic decompensation, hepatocellular carcinoma, and liver-related mortality [3].

With a global prevalence of 30%, NAFLD demonstrates rising incidence largely driven by increasing rates of obesity, insulin resistance, type 2 diabetes mellitus (T2DM), and metabolic syndrome [1]. Metabolic dysfunction plays a central role in the development of NAFLD, which is now widely understood as the liver manifestation of systemic metabolic disease. Reflecting this shift in understanding, an international consensus recently introduced updated terminology, replacing NAFLD with metabolic dysfunction-associated steatotic liver disease (MASLD) and NASH with metabolic dysfunction-associated steatohepatitis (MASH) [3]. Despite this transition, the term NAFLD remains common in earlier clinical trials and much of the existing literature.

For the purposes of this review, studies using the historical NAFLD/NASH terminology were included and interpreted within the current MASLD/MASH framework, where diagnostic definitions substantially overlapped. Terminology used in the original studies is retained when describing individual trial populations, while MASLD/MASH terminology is used when discussing the broader contemporary disease framework.

Apart from liver-specific consequences, NAFLD is linked to a broad range of extrahepatic complications, including higher risks of cardiovascular disease, chronic kidney disease, and overall mortality [4]. Among all disease features, fibrosis stage consistently emerges as the strongest predictor of long-term outcomes, underscoring the need for timely identification and intervention before progression to advanced disease [5].

### 1.2. Current Treatment Limitations

Despite the rising prevalence and clinical impact of NAFLD, effective treatment options remain limited. Management still relies heavily on lifestyle modification, including dietary changes, increased physical activity, and weight reduction. Achieving and maintaining a weight loss of around 7–10% has been shown to improve steatosis, inflammation, and key histological features of NASH, with even greater reductions potentially improving fibrosis [6]. However, sustaining these lifestyle changes over the long term is challenging, and many patients struggle to reach or maintain clinically meaningful weight loss.

Pharmacological options remain limited, and there is still no universally recommended first-line medication for NAFLD across all patient groups. Pioglitazone has shown histological benefit in patients with biopsy-proven NASH, particularly those with type 2 diabetes, although concerns about weight gain, edema, fracture risk, and long-term safety have restricted its wider use [3]. Vitamin E has also demonstrated benefit in selected nondiabetic individuals with NASH, but its role remains debated due to uncertainties about long-term adverse effects and questions about how well the findings generalize to broader patient populations [3,5].

Bariatric surgery can lead to meaningful improvements in steatosis and fibrosis among carefully selected individuals with severe obesity, but its invasive nature limits its suitability as a population-wide strategy [7]. As a result, there remains a clear unmet need for therapies that effectively target both metabolic dysfunction and liver-specific disease progression.

### 1.3. GLP-1 Receptor Agonists as a Potential Therapeutic Option

Glucagon-like peptide-1 receptor agonists (GLP-1 RAs) are well-established therapies for type 2 diabetes and obesity. Medications such as liraglutide, semaglutide, dulaglutide and exenatide improve glycemic control through several complementary mechanisms, including glucose-dependent enhancement of insulin secretion, suppression of inappropriate glucagon release, delayed gastric emptying, and satiety-mediated weight loss [8].

Their potential role in NAFLD/MASH has gained considerable attention because they target several mechanisms central to disease pathogenesis. Insulin resistance promotes impaired suppression of adipose tissue lipolysis, resulting in increased free fatty acid flux to the liver, enhanced de novo lipogenesis, hepatic triglyceride accumulation and steatosis [9]. By improving insulin sensitivity, enhancing glycemic control, reducing appetite and promoting weight loss, GLP-1 receptor agonists may reduce the metabolic substrate burden that contributes to hepatic fat accumulation and disease progression [8,9]. In addition, reductions in adiposity and improvements in systemic metabolic dysfunction may help attenuate downstream inflammatory and lipotoxic pathways that contribute to the progression from steatosis to steatohepatitis [9].

Beyond these systemic metabolic effects, GLP-1 receptor agonists may influence hepatic lipid metabolism more directly by reducing lipogenesis and promoting fatty acid oxidation, thereby limiting intrahepatic lipid deposition [10,11]. Excess lipid accumulation contributes to lipotoxicity, which in turn promotes oxidative stress, endoplasmic reticulum stress and hepatocyte injury [9]. Oxidative stress generated by excess reactive oxygen species is believed to play a key role in the progression from simple steatosis to steatohepatitis and fibrosis [9]. Preclinical and mechanistic studies suggest that GLP-1 receptor agonists may attenuate oxidative injury and reduce lipid-induced cellular stress, thereby limiting hepatocellular damage [10,11,12].

Inflammatory signaling also represents an important therapeutic target. Hepatic lipid accumulation activates inflammatory pathways involving Kupffer cells, macrophages, and pro-inflammatory cytokines, contributing to hepatocyte injury and fibrogenesis [9,10,11]. GLP-1 RAs have been associated with reductions in inflammatory signaling and markers of hepatic inflammation, which may partially explain their beneficial effects on steatohepatitis [10,11]. Clinical improvements in steatohepatitis resolution observed in LEAN and the phase 2 semaglutide trial are consistent with this broader anti-inflammatory and metabolic effect, although they do not prove a direct anti-inflammatory mechanism [13,14].

Mitochondrial dysfunction is increasingly recognized as a critical feature of NAFLD progression [9,12]. Impaired mitochondrial β-oxidation and altered energy homeostasis promote the generation of reactive oxygen species, cellular injury and further lipid accumulation. Experimental evidence suggests that GLP-1 RAs may improve mitochondrial function, enhance fatty acid oxidation and reduce mitochondrial stress, although direct confirmation of these effects in humans remains limited [10,11,12].

**Figure 1:**
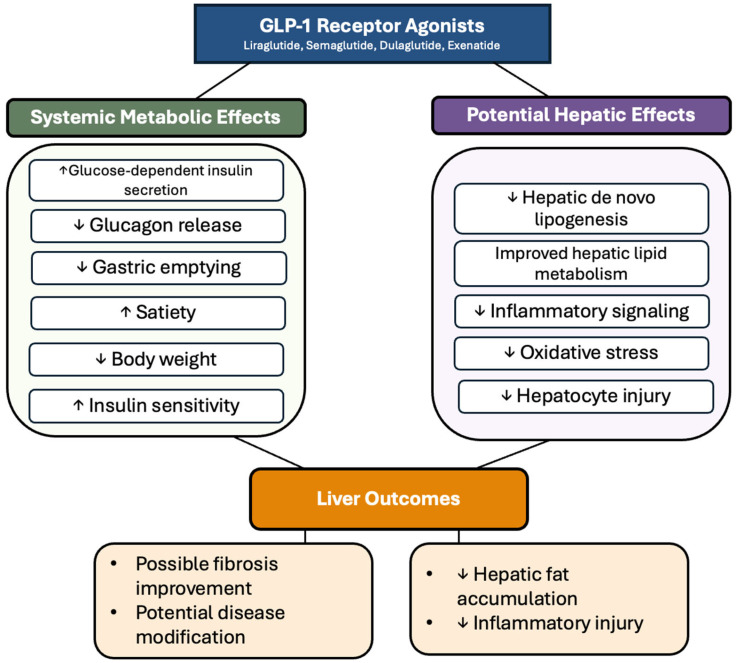
Mechanisms of GLP-1 Receptor Agonist Benefit in NAFLD/MASH. ↑ indicates an increase or enhancement of a biological process; ↓ indicates a decrease or suppression of a biological process. Arrows denote proposed mechanistic links between GLP-1 receptor agonist activity and liver outcomes.

Overall, the benefits of GLP-1 RAs in NAFLD/MASH are likely mediated through both weight-loss-dependent and weight-loss-independent mechanisms [6,8,9,10,11,12]. While improvements in adiposity and insulin resistance remain major drivers of therapeutic benefit, additional effects on hepatic lipid metabolism, oxidative stress, inflammatory signaling and mitochondrial function may contribute to their hepatoprotective properties. The proposed mechanisms through which GLP-1 receptor agonists may improve NAFLD/MASH are summarized in Figure 1.

### 1.4. Existing Evidence and Knowledge Gap

Several randomized controlled trials (RCTs) and observational studies have examined the effects of GLP-1 RAs in patients with NAFLD and NASH, assessing outcomes such as liver fat content, liver enzyme levels, fibrosis markers, histological changes, and safety profiles. Early evidence from the Liraglutide Efficacy and Action in NASH (LEAN) trial showed that liraglutide was associated with higher rates of NASH resolution compared with placebo [13]. More recent studies evaluating semaglutide have reported similarly encouraging improvements in NASH resolution, although effects on fibrosis have been less consistent across trials [14].

Despite these promising findings, the current evidence base remains heterogeneous. Studies differ widely in design, sample size, follow-up duration, patient characteristics, diagnostic approaches, and the specific GLP-1 RA agents evaluated [15,16]. Some rely on imaging-based measures of liver fat, whereas others use histological endpoints or surrogate biomarkers. Additionally, many trials include individuals with obesity or type 2 diabetes, which can make it challenging to isolate treatment effects in broader NAFLD populations [17,18].

Although previous reviews have summarized pharmacologic therapies for NAFLD more generally, an updated synthesis focused specifically on GLP-1 RAs is needed. The emergence of newer trials, evolving disease terminology, and growing clinical interest in these agents further underscore the importance of a dedicated evaluation. Accordingly, this systematic review aims to assess the therapeutic effects of GLP-1 receptor agonists on liver-related outcomes in patients with NAFLD.

## 2. Methods

### 2.1. Study Design and Information Sources

This systematic review adhered to the Preferred Reporting Items for Systematic Reviews and Meta-Analyses (PRISMA) 2020 guidelines, and the completed PRISMA 2020 checklist is provided as Supplementary Table S5. A predefined protocol was registered with the International Prospective Register of Systematic Reviews (PROSPERO; CRD420261337353). A comprehensive literature search was performed across PubMed (MEDLINE), Scopus, Embase, and the Cochrane Library.

### 2.2. Eligibility Criteria and Study Selection Framework

Eligibility was defined using the Population, Intervention, Comparator, Outcomes, and Study design (PICOS) framework (Table 1). Searches used controlled vocabulary and keywords for GLP-1 receptor agonists and non-alcoholic fatty liver disease. Searches were limited to human studies published in English from 1 January 2000 to 1 April 2026. A detailed eligibility criteria can be found in Table 1.

### 2.3. Search String

The search string can be found in Supplementary Table S1. Database-specific strategies were applied.

### 2.4. Study Selection

**Figure 2:**
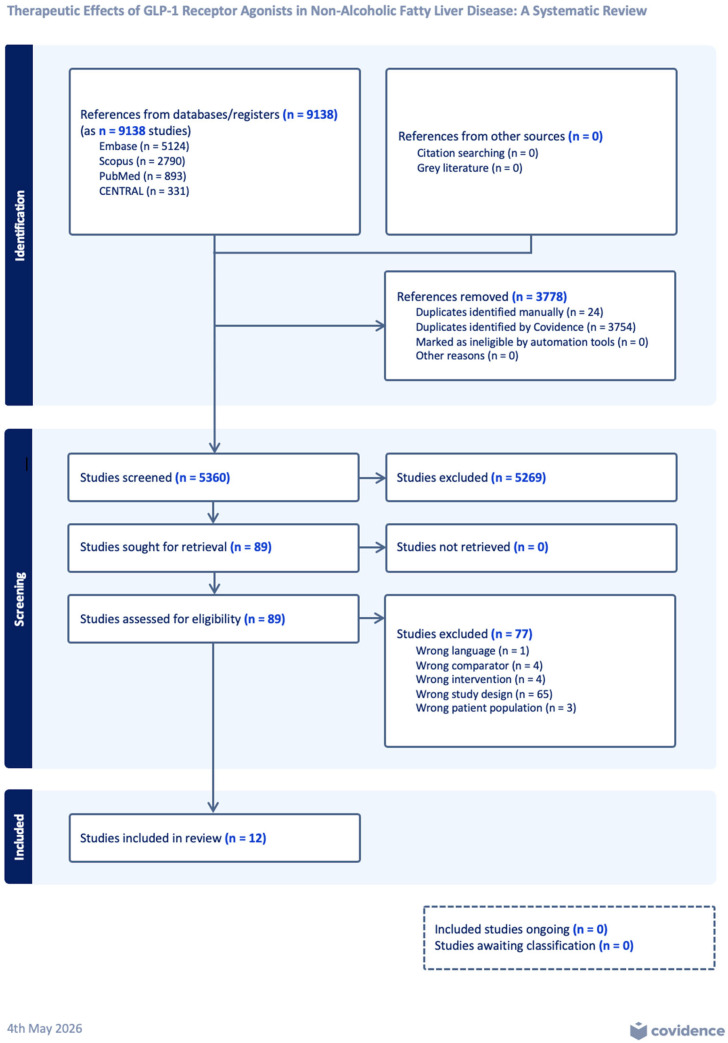
PRISMA Flow Diagram.

Study selection was conducted using Covidence (Veritas Health Innovation, Melbourne, Australia), a web-based systematic review management platform. Following duplicate removal, two reviewers independently screened titles and abstracts according to the eligibility criteria. Subsequently, two additional reviewers independently assessed the relevant studies at the full-text stage. At the title and abstract stage, records were excluded if they clearly did not meet the predefined criteria for population, intervention, outcome, or study design. At the full-text stage, exclusions were based on the PICOS framework, including ineligible population, absence of a GLP-1 receptor agonist intervention, lack of liver-specific outcomes, inability to isolate the effect of GLP-1 receptor agonist therapy, or ineligible study design. Disagreements at any stage were resolved through discussion, with consultation of a third reviewer when required. Reasons for exclusion at the full-text stage were documented, and the selection process was illustrated using a PRISMA flow diagram (Figure 2). The PRISMA flow diagram summarizes the number of records identified, duplicates removed, records screened, full-text articles assessed, full-text exclusions with reasons, and final studies included in the review.

### 2.5. Data Extraction

Data extraction was carried out in Covidence using a standardized form. Two reviewers independently extracted data from each included study. Extracted information includes study details, design, sample size, participant characteristics, intervention and comparator groups, outcomes reported, main results, and any adverse events. Any discrepancies between reviewers were resolved through discussion, with a third reviewer consulted if necessary.

### 2.6. Risk of Bias Assessment

Risk of bias was assessed independently by two reviewers for all included studies. Randomized controlled trials were evaluated using the Cochrane Risk of Bias 2 (RoB 2) tool, while observational studies were assessed using the Newcastle–Ottawa Scale. Any differences in assessment were discussed and resolved between the reviewers, with a third reviewer consulted if needed.

### 2.7. Certainty of Evidence

Certainty of evidence for the main outcomes was assessed using the Grading of Recommendations Assessment, Development, and Evaluation (GRADE) approach. This involved considering factors such as risk of bias, consistency of results across studies, directness of the evidence, precision of estimates, and potential publication bias. Based on these domains, the overall certainty of evidence was rated as high, moderate, low, or very low.

### 2.8. Data Synthesis

Data synthesis was conducted by summarizing the findings of the included studies based on intervention type and reported outcomes. The feasibility of conducting a meta-analysis was assessed in consultation with a data analyst and deemed inappropriate due to differences in study design, populations, and outcome reporting. As a result, the findings are presented narratively, with results grouped and compared across studies to highlight overall trends and key differences.

## 3. Results

After removing duplicates and completing screening, 12 studies met the inclusion criteria. A summary of the characteristics of the included studies is presented in Table 2.

### 3.1. Liver Fat

Across the included studies, liver fat was the outcome that improved most consistently with glucagon-like peptide-1 receptor agonist (GLP-1 RA) therapy. In the Semaglutide Treatment Effect in People with Obesity and Non-Alcoholic Fatty Liver Disease Using Magnetic Resonance Imaging-Based Assessment of Liver Fat (SAMARA) trial, semaglutide led to a higher proportion of patients achieving at least a 30% reduction in magnetic resonance imaging–proton density fat fraction (MRI-PDFF) compared with placebo (60% vs. 17%, *p* = 0.047) [19]. The mean percentage reduction in MRI-PDFF was also greater with semaglutide (−35% vs. −17%), although this comparison was not statistically significant [19].

Similar findings were reported in the Dulaglutide Liver Fat (D-LIFT) trial, where dulaglutide produced a control-corrected absolute reduction in liver fat of −3.5% and a relative reduction of −26.4% compared with control [15]. Beinaglutide also lowered intrahepatic triglyceride (IHTG) content compared with lifestyle intervention alone, with patients in the beinaglutide group being more likely to achieve a ≥50% reduction in IHTG content (OR 4.35, *p* = 0.028) [22]. Together, these studies show a consistent direction of effect across semaglutide, dulaglutide, and beinaglutide, although comparator groups differed between placebo, standard care and lifestyle intervention [15,19,22].

A similar trend was seen with liraglutide. In Khoo et al., the liver fat fraction decreased significantly during liraglutide treatment (−7.0 ± 7.1%) and was comparable to the reduction observed with structured diet and exercise (−8.1 ± 13.2%) [23,24]. Zhang et al. additionally reported that liraglutide reduced hepatic fat content on proton magnetic resonance spectroscopy (^1^H-MRS) from 24.1 ± 3.0 to 20.1 ± 3.8, whereas the pioglitazone group showed a smaller reduction from 23.9 ± 3.8 to 22.4 ± 3.5 [28]. The liraglutide studies, therefore, also supported reductions in hepatic fat, but the magnitude of benefit varied by comparator and study design [23,28]. In patients with non-alcoholic steatohepatitis (NASH)-related cirrhosis, semaglutide reduced MRI-PDFF, with 49% of semaglutide-treated patients achieving a ≥30% reduction in steatosis compared with 13% of placebo-treated patients [25].

Overall, reductions in liver fat were reported across multiple GLP-1 RAs and imaging methods. The most consistent evidence came from studies using quantitative imaging-based liver fat measures, while differences in comparator groups, sample sizes, and baseline disease stages limited direct comparisons between agents.

### 3.2. Liver Enzymes

Liver enzyme changes were reported in several studies, although findings were less consistent than those for liver fat. The clearest response was seen in SAMARA, where semaglutide reduced alanine aminotransferase (ALT) by 31.8 U/L versus 8.6 U/L with placebo (*p* = 0.021), aspartate aminotransferase (AST) by 18.2 U/L versus 7.5 U/L (*p* = 0.017), and gamma-glutamyl transferase (GGT) by 15.6 U/L versus 0.6 U/L (*p* = 0.009) [19]. Albarmawi et al. reported a similar ALT response after six months of semaglutide treatment. ALT decreased by 19.4 U/L compared with 2.8 U/L in the comparator group, with a significant adjusted between-group difference of −15.9 U/L (*p* = 0.01) [20]. AST also decreased with semaglutide, although the between-group effect was less clear [20]. Together, these findings suggest that ALT improvement was most reproducible in the semaglutide studies, while AST responses were less consistent [19,20].

Findings for other GLP-1 RAs were more mixed. In D-LIFT, dulaglutide significantly reduced GGT compared with control (between-group difference −13.1 U/L, *p* = 0.025), but changes in AST and ALT did not reach statistical significance [15]. Liraglutide findings were also inconsistent. Khoo et al. reported reductions in ALT and AST alongside reduced liver fat, but these changes were similar to those observed with diet and exercise [24]. Zhang et al. found that liraglutide reduced hepatic fat without significantly improving liver enzymes [28]. Thus, compared with liver fat outcomes, enzyme responses varied more clearly by agent, comparator and enzyme measured [15,19,20,23,24,28].

Overall, ALT improved most often, particularly in semaglutide studies, while AST and GGT findings varied across agents and study designs. Liver enzyme changes, therefore, provided supportive evidence of treatment response, but they were less consistent than imaging-based liver fat reductions and were not uniformly observed across GLP-1 RA studies.

### 3.3. Liver Fibrosis

Fibrosis findings were less uniform than liver fat and enzyme outcomes. Across studies, fibrosis was assessed using different approaches, including biopsy-based fibrosis staging in randomized trials and non-invasive fibrosis scores in observational studies, which limits direct comparison between findings. In the LEAN trial, liraglutide did not significantly increase fibrosis improvement compared with placebo (26% vs. 14%, *p* = 0.46). However, worsening of fibrosis was less frequent with liraglutide than with placebo (9% vs. 36%, *p* = 0.04) [13].

The semaglutide trials showed mixed results. In the phase 2 trial, semaglutide improved NASH resolution, but fibrosis improvement was not significant. Fibrosis improvement without worsening NASH occurred in 43% of patients receiving semaglutide 0.4 mg compared with 33% receiving placebo (*p* = 0.48) [14]. In contrast, the Effect of Semaglutide in Subjects with Non-Cirrhotic Non-Alcoholic Steatohepatitis (ESSENCE) trial showed a significant fibrosis benefit, with fibrosis improvement without worsening steatohepatitis in 36.8% of semaglutide-treated patients compared with 22.4% of placebo-treated patients (*p* < 0.001) [26]. Thus, among biopsy-based semaglutide trials, fibrosis benefit was not significant in the earlier phase 2 study but was significant in the larger phase 3 ESSENCE trial in non-cirrhotic MASH with F2–F3 fibrosis [14,26].

Observational studies also reported fibrosis-related outcomes. Choi et al. reported lower progression to high-risk fibrosis-4 index (FIB-4) among GLP-1 RA users than dipeptidyl peptidase-4 (DPP-4) inhibitor users, with fibrosis progression rates of 3.25 versus 4.29 per 100 person-years (HR 0.75, 95% CI 0.65–0.87) [21]. Wood et al. similarly reported less FIB-4 worsening among GLP-1 RA users compared with controls [27]. These observational findings were directionally consistent with a protective association, but they relied on non-invasive fibrosis scores rather than biopsy-confirmed fibrosis regression [21,27].

In patients with NASH-related compensated cirrhosis, semaglutide did not improve fibrosis. Fibrosis improvement without worsening NASH occurred in 11% of semaglutide-treated patients and 29% of placebo-treated patients, with no significant difference (*p* = 0.087) [25]. This contrasted with the fibrosis benefit observed in non-cirrhotic F2–F3 MASH in ESSENCE, suggesting that fibrosis outcomes differed by baseline disease stage [25,26].

Overall, fibrosis outcomes differed across trials, observational studies, and disease stages. The strongest evidence of fibrosis improvement was observed in non-cirrhotic MASH with F2–F3 fibrosis, whereas benefit was not demonstrated in established compensated cirrhosis.

### 3.4. Liver Stiffness

Liver stiffness findings were less consistent than liver fat or enzyme outcomes. Similar to liver fibrosis, stiffness was assessed using different non-invasive methods, including vibration-controlled transient elastography (VCTE) and magnetic resonance elastography (MRE), which limited direct comparison. In SAMARA, semaglutide did not significantly reduce stiffness measured by VCTE. The absolute change was −2.0 kPa with semaglutide compared with −1.81 kPa with placebo (*p* = 0.292), and the percentage change was also similar (−16% vs. −13%, *p* = 0.218) [19]. MRE likewise showed no significant treatment effect (*p* = 0.548) [19].

Similar findings were seen in other trials. In D-LIFT, liver stiffness decreased within the dulaglutide group from 10.8 to 9.3 kPa, but the between-group difference was not significant (−1.31 kPa, *p* = 0.123) [15]. Fan et al. also reported a trend toward improvement with beinaglutide, with stiffness improvement in 75% of patients compared with 42.9% in the lifestyle group, although this was not statistically significant (*p* = 0.145) [22].

Khoo et al. reported small within-group reductions in stiffness with both liraglutide (−0.25 ± 0.27 kPa, *p* = 0.007) and diet and exercise (−0.12 ± 0.19 kPa, *p* = 0.04) [24]. However, the between-group difference was not significant (*p* = 0.17). In Loomba et al.’s cirrhosis trial, semaglutide also did not significantly improve MRE-measured stiffness (ETR 0.93, *p* = 0.30) [25].

Overall, most between-group comparisons for liver stiffness were not statistically significant.

### 3.5. Histological Outcomes

Histological outcomes generally supported the benefits of GLP-1 RAs, especially in resolving steatohepatitis. In the LEAN trial, liraglutide significantly increased the proportion of patients achieving NASH resolution without worsening fibrosis compared with placebo [13].

Newsome et al. later reported a dose–response effect with semaglutide. In the phase 2 trial, the highest semaglutide dose achieved NASH resolution in 59% of patients compared with 17% in the placebo group [14]. Improvement in fibrosis stage did not differ significantly from placebo [14]. This pattern was similar to LEAN in showing benefit for steatohepatitis resolution, while fibrosis improvement remained less consistent [13,14].

The phase 3 ESSENCE trial reported significant improvements in both primary histological endpoints. Semaglutide significantly improved the resolution of steatohepatitis without worsening fibrosis, and reduced fibrosis without worsening steatohepatitis [26]. Compared with earlier phase 2 data, ESSENCE provided stronger histological evidence for fibrosis improvement in non-cirrhotic MASH with F2–F3 fibrosis [26].

In contrast, Loomba et al. found no significant histological improvement in patients with compensated NASH cirrhosis [25]. NASH resolution and fibrosis improvement were not significantly different between semaglutide and placebo [25].

Overall, histological outcomes varied across trials, with NASH resolution reported more consistently than fibrosis improvement.

### 3.6. Hepatic Biomarkers

Several studies reported favorable changes in hepatic biomarkers. These biomarkers reflected different aspects of liver injury and disease activity, including composite steatohepatitis risk, hepatocyte apoptosis, inflammation, and fibrogenesis. In SAMARA, semaglutide significantly reduced the FibroScan-AST (FAST) score compared with placebo [19].

Khoo et al. reported reductions in caspase-cleaved cytokeratin-18 (cCK-18), a marker of hepatocyte apoptosis [23]. Zhang et al. found that liraglutide significantly reduced fetuin-A levels [28]. Thus, liraglutide studies reported improvements in biomarkers of hepatocyte injury and metabolic liver dysfunction, although the measured biomarkers varied across studies [23,28].

In Loomba et al.’s cirrhosis trial, semaglutide significantly reduced N-terminal type III collagen pro-peptide (PRO-C3) and high-sensitivity C-reactive protein (hs-CRP), although enhanced liver fibrosis (ELF) score did not significantly differ between groups [25]. This pattern contrasted with the lack of significant improvement in histological fibrosis in the same cirrhosis trial, showing that changes in individual biomarkers were not always matched by improvements in broader fibrosis endpoints [25].

Overall, hepatic biomarker findings varied by study and biomarker assessed. Across studies, biomarker improvements were generally consistent with reduced hepatic injury, inflammation or fibrogenic activity, but direct comparisons were limited.

### 3.7. Adverse Events

Adverse events were mostly gastrointestinal, and serious liver-related safety issues were uncommon. In SAMARA, gastrointestinal events were reported in 50% of patients receiving semaglutide and 41% receiving placebo, with no significant difference between groups (*p* = 0.59) [19]. The reported symptoms included diarrhea (18% vs. 11%), nausea (16% vs. 11%), vomiting (12% vs. 3%), and reflux symptoms (22% vs. 9%) [19]. Only one serious adverse event occurred in the semaglutide group, a concussion that was judged unlikely to be related to treatment. No gallbladder-related events or pancreatitis were reported [19].

The gastrointestinal signal was more obvious in the liraglutide trials. In LEAN, gastrointestinal disorders occurred in 81% of patients receiving liraglutide compared with 65% receiving placebo [13]. Diarrhea was reported in 38% of liraglutide-treated patients, constipation in 27%, and reduced appetite in 31% [13]. Khoo et al. also reported frequent gastrointestinal symptoms with liraglutide, including nausea in 80%, abdominal discomfort or bloating in 100%, diarrhea in 33.3%, and flatulence in 40% [24]. One patient in that study stopped treatment because of recurrent injection-site reactions [24].

The other studies fit broadly with this safety profile. Fan et al. reported mainly mild-to-moderate adverse events with beinaglutide, mostly gastrointestinal, and these symptoms decreased over time [22]. In D-LIFT, dulaglutide was not linked to serious drug-related adverse events, although three patients discontinued treatment because of upper gastrointestinal symptoms [15].

In the semaglutide cirrhosis trial, adverse events were common in both groups, affecting 89% of semaglutide-treated patients and 79% of placebo-treated patients [25]. Nausea, diarrhoea, and vomiting were more frequent with semaglutide, occurring in 45%, 19%, and 17% of patients, respectively [25]. Hepatic and renal function remained stable, and no hepatic decompensation events or deaths occurred [25].

In ESSENCE, adverse events were also slightly more common with semaglutide than placebo (86.3% vs. 79.7%) [26]. Serious adverse events, however, were equal in both groups (13.4%), and discontinuation due to adverse events was low (2.6% with semaglutide vs. 3.3% with placebo) [26].

Overall, gastrointestinal symptoms were the most frequently reported adverse events across GLP-1 RA studies.

### 3.8. Risk of Bias and Certainty of Evidence

Risk of bias was assessed for all randomized and interventional studies using the Cochrane Risk of Bias 2 (RoB 2) tool. Overall, the randomization process was generally rated as low risk, while concerns were more commonly related to missing outcome data, deviations from intended interventions, outcome measurement, and selection of reported results. Full domain-level assessments are presented in Supplementary Table S2.

Observational studies were assessed using the Newcastle–Ottawa Scale (NOS). Overall study quality ranged from moderate to high. These studies provided real-world evidence, but their findings were based on non-randomized designs, and several relied on surrogate outcomes such as FIB-4. Full NOS ratings are presented in Supplementary Table S3.

Certainty of evidence varied across outcomes. Evidence was strongest for steatohepatitis resolution, which was rated as moderate to high, and for short-term common adverse events, if included in the Grading of Recommendations Assessment, Development and Evaluation (GRADE) assessment. Certainty for liver fat reduction was low to moderate, while certainty was low for liver enzyme changes, fibrosis improvement, liver stiffness, and hepatic biomarkers. The outcome-level GRADE assessment is summarized in Supplementary Table S4.

## 4. Discussion

### 4.1. Summary of Principal Findings

Overall, treatment with GLP-1 receptor agonists was associated with improvements in liver-related outcomes across several domains, with the most consistent benefits observed in markers of disease activity and steatohepatitis [13,14,19,26]. However, these effects were not uniform across all endpoints, with greater variability observed in outcomes reflecting more advanced disease, particularly fibrosis and cirrhosis [13,14,25]. Differences in study design, baseline population characteristics, and follow-up duration likely contributed to heterogeneity in the reported effects. Taken together, the current evidence supports a beneficial role for GLP-1 receptor agonists in modifying key features of NAFLD, especially in earlier stages of disease, while highlighting important limitations regarding their impact on more advanced fibrosis.

### 4.2. Effects on Liver Fat, Enzymes, and Biomarkers

The most consistent finding across the included studies was the reduction in liver fat. Several RCTs showed significant decreases in MRI-based measures such as MRI-PDFF, intrahepatic triglyceride content, and proton magnetic resonance spectroscopy-based hepatic fat content, with consistent findings across semaglutide, dulaglutide, liraglutide, and beinaglutide [15,19,22,23,24,25,26,28]. These improvements were demonstrated across different imaging techniques and patient populations, including those with advanced disease, supporting a class-wide effect on hepatic steatosis.

Improvements in liver enzymes, particularly ALT, were commonly observed. The most consistent evidence was reported in semaglutide trials, which showed significant between-group reductions in ALT, AST, and GGT [19,20]. Findings for other GLP-1 RAs were less consistent. Dulaglutide was associated with partial improvement, although this was not consistently statistically significant [15], while liraglutide studies showed reductions that were either comparable to lifestyle intervention or not statistically significant [23,24,28].

Although reductions in ALT, AST, and GGT may reflect improvement in hepatocellular injury or metabolic liver stress, they should not be interpreted as direct evidence of histological improvement. For example, in some semaglutide studies, improvements in liver enzymes and metabolic parameters were observed without significant fibrosis regression, particularly in the phase 2 semaglutide trial and the compensated cirrhosis trial [14,25]. Therefore, liver enzyme changes are best interpreted as supportive markers of treatment response rather than reliable surrogates for steatohepatitis resolution or fibrosis improvement.

Changes in hepatic biomarkers also supported a beneficial effect on liver pathology. Reductions in composite indices such as FAST, hepatocyte injury markers such as cytokeratin-18, metabolic liver biomarkers such as fetuin-A, and fibrogenesis markers such as PRO-C3 were reported across several studies [19,23,25,28]. These markers reflect different aspects of liver injury, apoptosis, inflammation, and fibrogenesis, but they were not assessed consistently across all studies. Therefore, biomarker improvements should be interpreted as supportive evidence of reduced disease activity rather than definitive evidence of histological improvement.

An important consideration is the extent to which the observed hepatic benefits are mediated by weight loss versus direct hepatoprotective effects of GLP-1 receptor agonists. Several included studies demonstrated parallel improvements in body weight, hepatic steatosis, liver enzymes and histological activity, suggesting that weight reduction is likely a major contributor to treatment response [6,22,24]. This interpretation is supported by studies such as Fan et al. and Khoo et al., in which improvements in hepatic fat content were closely associated with reductions in body weight [22,24]. However, based on the known biology of GLP-1 signaling and the central role of insulin resistance and lipotoxicity in NASH pathogenesis, GLP-1 receptor agonists may also exert effects beyond weight loss, including improvements in insulin sensitivity, hepatic lipid handling, oxidative stress, inflammatory signaling and mitochondrial function [8,9]. Histological improvements in steatohepatitis observed in trials such as LEAN, the phase 2 semaglutide study, and ESSENCE support a potential disease-modifying effect of GLP-1 receptor agonists [13,14,26]. Nevertheless, these clinical studies were not specifically designed to distinguish weight-dependent from weight-independent mechanisms, and the relative contribution of each pathway remains incompletely understood.

### 4.3. Effects on Fibrosis, Liver Stiffness, and Histology

Findings related to fibrosis were heterogeneous and appeared to vary according to disease stage and study design. Earlier randomized trials showed limited or non-significant improvement in fibrosis, despite clear benefit in steatohepatitis resolution [13,14]. In contrast, the larger phase 3 ESSENCE trial provided stronger evidence of a fibrosis benefit, with semaglutide significantly increasing the proportion of patients achieving fibrosis improvement without worsening steatohepatitis in non-cirrhotic MASH with F2–F3 fibrosis [26]. This remains the strongest evidence of a fibrosis effect among the included studies.

Observational studies also suggested a possible protective effect, with lower rates of fibrosis progression among users of GLP-1 receptor agonists than among controls [21,27]. These findings should be interpreted cautiously, however, because they relied on surrogate measures such as FIB-4 and were subject to the limitations inherent in non-randomized study designs.

Interpretation of fibrosis outcomes was complicated by differences in assessment methods. Biopsy provides direct histological staging but is limited by sampling variability, while elastography-based measures such as VCTE and MRE estimate liver stiffness and may be affected by inflammation, steatosis, congestion and technical factors. Serum-based indices such as FIB-4 are useful for risk stratification in large cohorts but do not directly measure fibrosis regression. Therefore, biopsy, elastography and serum biomarker outcomes should not be interpreted as equivalent endpoints [29,30].

Liver stiffness measurements showed modest and inconsistent changes across studies. While some within-group improvements were noted, most between-group comparisons did not reach statistical significance [15,19,22,24,25,28]. This inconsistency may reflect the limited sensitivity of stiffness measurements over short follow-up periods, differences in baseline fibrosis stage, or a delayed response between metabolic improvement and measurable changes in fibrotic burden.

Given these limitations, histological assessment remains a crucial metric for evaluating true disease modification. Randomized trials, including the LEAN study, the phase 2 semaglutide trial, and the ESSENCE trial, have consistently demonstrated that GLP-1 receptor agonists facilitate the resolution of steatohepatitis [13,14,26]. However, findings regarding fibrosis regression are less uniform and appear to be stage-dependent.

One possible explanation is that GLP-1 receptor agonists primarily target upstream metabolic and inflammatory drivers of MASH, including insulin resistance, adipose tissue dysfunction, hepatic steatosis and inflammatory signaling, which may be more readily reversible than established fibrosis. In advanced fibrosis and cirrhosis, extensive extracellular matrix deposition, architectural distortion, vascular remodeling and regenerative nodule formation may be less responsive to therapies that primarily improve metabolic dysfunction over relatively short trial durations [31]. This may explain why improvements in steatohepatitis, liver enzymes and hepatic fat content were observed more consistently than fibrosis regression across several studies. Notably, semaglutide has not demonstrated significant histological improvement in patients with established compensated NASH cirrhosis [25], suggesting that advanced fibrosis may be less responsive to therapy within standard trial durations. Supporting this interpretation, the compensated NASH cirrhosis trial demonstrated improvements in steatosis, liver enzymes and cardiometabolic parameters, but no corresponding improvements in biopsy-based fibrosis outcomes [25]. Collectively, these histological data mirror the limitations of surrogate imaging markers, underscoring the challenges of assessing and quantifying fibrosis regression in advanced disease stages.

### 4.4. Possible Mechanisms of Benefit

The therapeutic effects of GLP-1 receptor agonists in NAFLD appear to be multifactorial and closely tied to their metabolic mechanisms. Weight loss is a central factor, as consistent reductions in body weight across trials correlated with improvements in both hepatic steatosis and secondary metabolic parameters [13,19,22,23,24,28]. Given the robust link between weight reduction and histological resolution of steatohepatitis, weight loss likely acts as a primary driver of the clinical benefits observed in these studies [6]. This interpretation is further supported by evidence from lifestyle intervention and bariatric surgery studies, which have demonstrated that substantial weight reduction alone can improve steatosis and steatohepatitis, although reversal of advanced fibrosis remains less predictable [6,7].

Improvement in glycemic control also appears to play a meaningful role, particularly among individuals with type 2 diabetes. Several of the included studies predominantly enrolled T2DM populations, and improvements in metabolic parameters were frequently observed alongside liver-specific outcomes. By reducing insulin resistance and promoting more efficient glucose handling, GLP-1 receptor agonists may attenuate hepatic de novo lipogenesis and lower the flux of free fatty acids to the liver, thereby reducing hepatic fat accumulation [9,32,33].

Beyond these systemic metabolic effects, several studies indicate that GLP-1 receptor agonists may also exert benefits within the liver that are not entirely dependent on weight loss. Improvements in biomarkers associated with hepatocyte injury, inflammation, and fibrogenesis, including cytokeratin-18, PRO-C3, and various inflammatory markers, suggest that these agents may influence intrahepatic pathways contributing to disease progression. This pattern may explain why improvements in steatohepatitis were observed even when changes in fibrosis were less consistent [19,23,25,28,32,33].

### 4.5. Safety and Tolerability

Across the included studies, GLP-1 receptor agonists were generally well tolerated in patients with NAFLD, MASH, and related metabolic diseases. Gastrointestinal adverse events, including nausea, vomiting, diarrhea, constipation, reflux symptoms, bloating, abdominal discomfort, and reduced appetite, were reported most frequently [13,14,19,23,24,25,26]. These events were typically mild to moderate in severity and rarely resulted in treatment discontinuation, although gastrointestinal intolerance was a clinically relevant cause of withdrawal in some studies. This adverse event profile aligns with the established pharmacological effects of GLP-1 RAs on gastric emptying, appetite, and gastrointestinal motility [8]. Consequently, gastrointestinal intolerance appears to be the principal limitation to treatment adherence rather than serious treatment-related toxicity.

The semaglutide studies provided a broadly reassuring safety profile. Although gastrointestinal symptoms occurred more frequently with semaglutide than placebo in several trials, discontinuation rates remained low, and serious adverse events were generally comparable between treatment groups [14,19,25,26]. Pancreatitis and gallbladder-related adverse events were reported infrequently across the included studies, and no consistent signal for increased pancreatitis risk was identified [13,14,19,25,26]. However, some semaglutide trials reported higher rates of gallbladder-related events and increases in pancreatic enzyme levels compared with placebo, suggesting that these remain relevant adverse events to monitor [14]. In the compensated NASH cirrhosis trial, hepatic and renal function remained stable, with no hepatic decompensation events or deaths reported during follow-up [25].

The liraglutide studies reported a notable gastrointestinal adverse-event burden, particularly in LEAN and Khoo et al. [13,23,24]. Nevertheless, these events were generally manageable and did not indicate a consistent pattern of serious treatment-related harm. Findings from the dulaglutide and beinaglutide studies were similarly consistent with the broader GLP-1 receptor agonist safety profile, with predominantly gastrointestinal adverse events and few serious drug-related concerns [15,22].

Overall, the available evidence suggests that GLP-1 receptor agonists have an acceptable safety and tolerability profile in NAFLD and MASH populations. Across the included studies, there was no consistent signal for pancreatitis, hepatic decompensation, treatment-related liver injury, or other major safety concerns [19,25,26]. However, interpretation of these findings is limited by the relatively short duration of most studies and the limited number of patients with advanced fibrosis or cirrhosis. Further research is needed to better define long-term tolerability, uncommon adverse events, gallbladder-related complications, treatment adherence and safety in patients with advanced liver disease.

### 4.6. Clinical Implications

The findings of this review suggest that GLP-1 receptor agonists may have a clinically useful role in the management of NAFLD/MASLD and MASH, particularly in patients with obesity, type 2 diabetes, or broader metabolic dysfunction. Their value lies in targeting key upstream drivers of disease progression, including excess adiposity, insulin resistance, and hepatic steatosis. Across the included studies, reduction in liver fat emerged as the most consistent liver-related benefit, suggesting that GLP-1 receptor agonists may be especially relevant for patients in whom hepatic steatosis and metabolic risk are dominant features [15,19,22,23,24,25,28].

Beyond their effects on liver-related outcomes, GLP-1 receptor agonists may offer important cardiovascular benefits in patients with NAFLD/MASLD. Cardiovascular disease remains the leading cause of morbidity and mortality in this population, reflecting the shared contributions of obesity, insulin resistance, dyslipidemia, hypertension and systemic inflammation [4]. Several GLP-1 receptor agonists have demonstrated reductions in major adverse cardiovascular events in patients with type 2 diabetes and high cardiovascular risk, while also promoting weight loss and improving glycemic control [34,35]. Although the studies included in this review primarily focused on hepatic outcomes rather than cardiovascular endpoints, the ability of GLP-1 receptor agonists to target both liver disease and cardiometabolic risk factors may represent an important therapeutic advantage. This dual benefit is particularly relevant given that many patients with MASLD experience cardiovascular complications before developing liver-related events. Future studies should further evaluate whether improvements in hepatic disease activity translate into reductions in long-term cardiovascular morbidity and mortality.

In clinical practice, improvements in liver enzymes and non-invasive biomarkers may provide supportive evidence of treatment response but should be interpreted with caution. Changes in ALT, AST, GGT, FAST score, cytokeratin-18, PRO-C3, or other biomarkers do not necessarily confirm histological improvement, particularly fibrosis regression [14,19,25]. Therefore, GLP-1 receptor agonist therapy should not replace appropriate fibrosis risk stratification, longitudinal monitoring, or specialist referral when advanced liver disease is suspected.

Current evidence suggests that GLP-1 receptor agonists may be most beneficial in patients with active steatohepatitis before cirrhosis becomes established. Their most consistent effects have been observed in reducing hepatic steatosis and promoting steatohepatitis resolution, whereas fibrosis improvement appears less consistent and may depend on baseline disease stage [13,14,26]. The strongest evidence for fibrosis benefit has been reported in patients with non-cirrhotic MASH and F2–F3 fibrosis, while evidence supporting reversal of established cirrhosis remains limited [25,26]. Consequently, GLP-1 receptor agonists should currently be viewed primarily as metabolic and steatohepatitis-directed therapies with potential disease-modifying effects rather than established antifibrotic treatments for advanced cirrhosis.

An important consideration is that many of the included studies enrolled patients with obesity, type 2 diabetes, or both, reflecting the populations in which GLP-1 receptor agonists are most commonly prescribed. Consequently, some of the observed benefits may be mediated through improvements in weight, insulin resistance and glycemic control. However, studies involving non-diabetic populations also demonstrated reductions in hepatic steatosis and improvements in liver-related outcomes, suggesting that the therapeutic effects of GLP-1 receptor agonists may extend beyond glycemic control alone [13,23,24]. Nevertheless, evidence in exclusively non-diabetic NAFLD/MASLD populations remains comparatively limited, and further studies are needed to determine whether treatment effects are of similar magnitude to those observed in patients with obesity and type 2 diabetes.

Overall, GLP-1 receptor agonists are best considered as part of a broader management strategy that includes lifestyle modification, weight reduction, glycemic control, cardiovascular risk reduction, and ongoing assessment of liver disease severity. Based on current evidence, the patients most likely to benefit are those with NAFLD/MASLD or non-cirrhotic MASH in the setting of obesity, type 2 diabetes, or metabolic dysfunction, whereas their role in advanced fibrosis and cirrhosis remains to be fully defined through longer-term studies.

### 4.7. Strengths and Limitations of the Evidence

This review has several strengths. It incorporated a diverse range of study designs, including randomized controlled trials, open-label studies, pilot trials, and retrospective cohort studies, allowing consideration of both controlled trial evidence and real-world data. Several included studies were double-blind, placebo-controlled randomized trials, strengthening the overall quality of evidence. The review also evaluated multiple GLP-1 receptor agonists, including semaglutide, liraglutide, dulaglutide, and beinaglutide, enabling comparison across agents within the same therapeutic class. Furthermore, the included studies assessed a broad range of liver-related outcomes, including imaging-based measures of hepatic steatosis, liver enzymes, fibrosis markers, liver stiffness, histological endpoints, hepatic biomarkers, and adverse events. This comprehensive assessment is particularly important because NAFLD/MASLD and MASH are multifaceted diseases that cannot be adequately evaluated using a single outcome measure.

Despite these strengths, several limitations of the evidence base should be acknowledged. First, sample size varied substantially across studies, ranging from small pilot and open-label trials to large phase 2 and phase 3 semaglutide studies [13,14,15,19,22,24,26]. Although observational cohorts provided larger real-world populations, they were more susceptible to confounding and incomplete outcome availability. For example, although Albarmawi et al. identified more than 4000 semaglutide users and 168,000 comparators, liver enzyme analyses were based on only a small subset of patients with available laboratory data, limiting the precision of those estimates [20].

Second, study duration varied considerably, ranging from 24 weeks or approximately 6 months to 72 weeks in the major histology trials. While shorter follow-up periods may be sufficient to detect changes in hepatic steatosis, body weight, glycemic control and liver enzymes, they may be inadequate to demonstrate meaningful fibrosis regression, which likely requires longer treatment exposure and observation [14,19,25,26].

Third, fibrosis assessment methods were heterogeneous. Some studies used paired liver biopsies with NASH Clinical Research Network/Kleiner fibrosis staging, whereas others relied on non-invasive measures such as VCTE, magnetic resonance elastography, FIB-4, FAST score, ELF score, PRO-C3 or NAFLD Fibrosis Score [13,14,19,21,25,26,27]. These approaches are clinically useful but not directly interchangeable. Biopsy directly evaluates histological fibrosis, whereas elastography and serum-based tools estimate liver stiffness or fibrosis risk and may be influenced by inflammation, steatosis, body habitus, age, platelet count and other metabolic factors.

Finally, diagnostic criteria differed across studies. Some enrolled patients with biopsy-confirmed NASH/MASH had defined fibrosis stages, whereas others included imaging-defined NAFLD, non-invasive test-defined at-risk MASH, or diagnosis-code-based MASLD/MASH cohorts [13,14,15,19,20,21,22,23,24,25,26,27,28]. In addition, the included studies spanned a period during which disease nomenclature evolved from NAFLD/NASH to MASLD/MASH. Although these classifications overlap substantially, the newer terminology places greater emphasis on underlying metabolic dysfunction and may not align perfectly with historical definitions. Race, ethnicity, and body composition were also reported inconsistently across studies, limiting detailed assessment of whether treatment effects differ across populations, including lean MASLD/MASH phenotypes. Collectively, these sources of clinical and methodological heterogeneity limit direct comparability between studies and support the decision to synthesize the findings narratively rather than perform a meta-analysis.

### 4.8. Future Directions

Future studies should focus on the long-term effects of GLP-1 receptor agonists on fibrosis progression and clinically meaningful liver outcomes. Although reductions in hepatic steatosis were observed consistently across the included studies, fibrosis findings were less uniform. ESSENCE demonstrated significant fibrosis improvement in patients with non-cirrhotic MASH and F2–F3 fibrosis [26], whereas earlier studies reported weaker or non-significant fibrosis effects [13,14]. In contrast, semaglutide did not significantly improve fibrosis or NASH resolution in patients with compensated NASH cirrhosis [25]. Consequently, longer and larger studies are needed to determine whether GLP-1 receptor agonists can prevent progression to cirrhosis or reduce hepatic decompensation, hepatocellular carcinoma, liver transplantation, and liver-related mortality.

Future research should also clarify which patient subgroups are most likely to benefit from GLP-1 receptor agonist therapy. The included studies involved heterogeneous populations, including patients with NAFLD, NASH, MASLD, MASH, obesity, type 2 diabetes, and compensated cirrhosis. Further studies should examine whether treatment response differs according to diabetes status, BMI, fibrosis stage, baseline liver fat burden, age, sex, ethnicity, or degree of insulin resistance. Such data would help identify the patients most likely to derive meaningful hepatic and metabolic benefit.

An additional area requiring investigation is the potential influence of ethnicity and body composition on treatment response. The clinical phenotype of MASLD/MASH differs across populations, and in East Asian populations a substantial proportion of patients develop MASLD/MASH despite not meeting conventional obesity criteria, a phenotype often referred to as lean MASLD/MASH [36,37,38,39]. Because many of the included studies enrolled individuals with obesity and/or type 2 diabetes, the generalizability of these findings to lean MASLD/MASH populations remains uncertain [37]. Future studies should evaluate whether the magnitude of benefit and underlying mechanisms of GLP-1 receptor agonists differ according to ethnicity, adiposity and metabolic phenotype.

The future management of MASLD/MASH will likely involve combination therapy rather than reliance on a single therapeutic agent. Given that disease progression is driven by interconnected metabolic, inflammatory, lipotoxic and fibrotic pathways, it is unlikely that any single treatment will adequately address all aspects of disease pathogenesis. While GLP-1 receptor agonists appear particularly effective for reducing body weight, improving insulin resistance, decreasing hepatic steatosis, and promoting steatohepatitis resolution, their effects on fibrosis have been less consistent [13,14,25,26]. Other emerging therapeutic approaches, including SGLT2 inhibitors, FXR agonists, thyroid hormone receptor-β agonists and dual incretin agonists, target complementary mechanisms and may ultimately serve synergistic rather than competing roles [40,41,42,43]. Future studies should compare the relative efficacy of these agents and determine whether combination approaches can produce greater improvements in fibrosis, long-term liver outcomes and overall cardiometabolic health than monotherapy alone.

The relationship between weight loss and liver improvement also warrants further investigation. Bariatric surgery provides an informative comparison because it produces substantially greater weight loss than currently achievable with GLP-1 receptor agonists and has demonstrated significant benefits in hepatic steatosis and steatohepatitis [44,45]. However, even bariatric surgery appears less effective in reversing advanced fibrosis or cirrhosis, suggesting that established fibrotic remodeling may be difficult to reverse despite substantial metabolic improvement [46]. As GLP-1 receptor agonists are increasingly used in patients with persistent obesity or metabolic dysfunction following bariatric surgery, future studies should evaluate whether combined surgical and pharmacological approaches provide additional benefit in patients with severe obesity and MASH [47,48].

## 5. Conclusions

This systematic review suggests that GLP-1 RAs offer meaningful therapeutic benefits in patients with NAFLD, MASH, and related metabolic dysfunction, particularly among individuals with obesity or type 2 diabetes. Across the included studies, the most consistent effect was a reduction in hepatic fat, supported by improvements across different GLP-1 receptor agonists and imaging methods. Liver enzymes and hepatic biomarkers also generally improved, although these findings were less consistent and should be interpreted as supportive rather than definitive evidence of histological improvement.

Histological outcomes suggest that GLP-1 receptor agonists may be most beneficial in patients with active steatohepatitis before advanced cirrhosis develops. Evidence for fibrosis improvement was promising, particularly in non-cirrhotic MASH with F2–F3 fibrosis, but remained inconsistent across earlier trials and studies involving compensated cirrhosis. Therefore, while GLP-1 RAs may have disease-modifying potential, their effects on advanced fibrosis and long-term liver-related outcomes require further investigation.

Overall, GLP-1 RAs appear to be a promising therapeutic option as part of a broader management strategy targeting metabolic dysfunction, hepatic steatosis, and steatohepatitis. They were generally well tolerated across the included studies, with gastrointestinal symptoms being the most common adverse events, and serious treatment-related liver safety concerns were uncommon. Future long-term studies are needed to clarify their role in preventing fibrosis progression, cirrhosis, hepatic decompensation, and liver-related mortality.

## Figures and Tables

**Table 1:** Inclusion and Exclusion Criteria.

PICO Component	Inclusion Criteria	Exclusion Criteria
Population (P)	1—Adults aged 18 years or older 2—Participants with a confirmed diagnosis of NAFLD, NASH, MASLD or MASH, established via liver biopsy, validated imaging modalities (e.g., MRI-PDFF, VCTE), or standardized non-invasive biomarker panels (e.g., FIB-4) accompanied by clinical assessment. 3—Comorbid obesity, pre-diabetes, T2DM allowed	1—Pediatric populations 2—Alcohol-related liver disease 3—Viral hepatitis, autoimmune liver disease, haemochromatosis, Wilson disease, drug-induced liver injury, or other secondary causes of hepatic steatosis.
Intervention (I)	1—Studies evaluating a GLP-1 receptor agonist as the primary intervention, including semaglutide, liraglutide, dulaglutide, exenatide, beinaglutide, or other agents. 2—Any dose, route, or treatment duration was eligible if liver-specific outcomes were reported before and after treatment or compared with a control group.	1—Studies evaluating medications that do not act on the GLP-1 receptor pathway 2—Studies of dual or triple incretin agonists, such as tirzepatide or retatrutide, unless GLP-1 receptor agonist effects were reported separately. 3—Combination therapies where the independent effect of GLP-1 receptor agonists cannot be determined
Comparator (C)	1—Placebo 2—Standard clinical care (e.g., routine management of comorbidities such as type 2 diabetes or obesity) 3—Lifestyle interventions (structured and supervised programs involving caloric restriction, specific dietary modifications, and/or exercise regimens) 4—Active pharmacological comparators (e.g., Pioglitazone, DPP-4 inhibitors) provided their specific effects can be isolated from the GLP-1 RA	Studies without a comparator group or baseline outcome reporting
Outcomes (O)	Studies reporting at least one primary or secondary hepatic efficacy outcome, such as: 1—Liver fat fraction or content via validated imaging (e.g., MRI-PDFF, H-MRS, or VCTE-CAP). 2—Histological changes via liver biopsy (e.g., steatohepatitis resolution, NAFLD Activity Score). 3—Quantitative changes in liver enzymes (ALT, AST, or GGT). 4—Liver fibrosis stage or stiffness assessed via biopsy, elastography (e.g., MRE, VCTE), or validated non-invasive biomarkers (e.g., FIB-4).	Studies reporting only metabolic outcomes (e.g., reductions in HbA1c, total body weight, or serum lipid profiles) without concurrently reporting at least one predefined liver-specific endpoint (e.g., changes in hepatic steatosis, transaminases, liver stiffness, or histology).
Study Characteristics (S)	1—Randomized controlled trials 2—Prospective cohort studies 3—Retrospective cohort studies	1—Case reports 2—case series 3—Editorials or commentaries 4—Animal or laboratory studies

**Table 2:** Study Characteristics of Included Studies.

Study (Author, Year)	Design	Country	Ethnicity	Population	Sample Size	Intervention	Comparator	Follow-up Duration	Outcomes
Newsome et al., 2021 [14]	Phase 2 RCT, double-blind, placebo-controlled	Multinational	Mixed; predominantly White, with Asian participants included	Biopsy-confirmed NASH (F1–F3)	320	Semaglutide 0.1, 0.2, or 0.4 mg once daily	Placebo	72 weeks	NASH resolution; fibrosis; ALT/AST; adverse events
Ajmera et al., 2026 [19]	RCT, double-blind, placebo-controlled	United States	Mixed; White, Hispanic, Asian, and Black participants reported	MASLD with BMI ≥ 27 or T2DM/prediabetes	55	Semaglutide 2.4 mg weekly	Placebo	52 weeks	Liver fat (MRI-PDFF); ALT/AST; weight/metabolic outcomes; adverse events
Albarmawi et al., 2025 [20]	Retrospective cohort study	United States	Mixed; White, Hispanic, Black, Asian, Other/Unknown reported	Overweight/obese adults with MASH	172,408	Semaglutide 2.4 mg weekly	No anti-obesity medication comparator	6 months	ALT/AST change; liver enzyme normalization
Armstrong et al., 2016 [13]	Phase 2 RCT, double-blind, placebo-controlled	United Kingdom	Not reported	Biopsy-proven NASH ± T2DM	52	Liraglutide 1.8 mg daily	Placebo	48 weeks treatment + 12 weeks follow-up	NASH resolution; fibrosis; ALT/AST; CK-18; weight/metabolic outcomes; adverse events
Choi et al., 2025 [21]	Retrospective cohort study (PSM)	United States	Mixed; predominantly White, with Black, Asian, and Other groups reported	MASLD + T2DM (FIB-4 < 2.67)	4476 matched (2238 vs. 2238)	GLP-1RA (various agents)	DPP-4 inhibitor	Follow-up until outcome, death, or 30 June 2024	Fibrosis progression (FIB-4); liver outcomes; adverse events
Fan et al., 2024 [22]	RCT, open-label	China	Chinese	T2DM + NAFLD	50	Beinaglutide 0.1 mg three times daily	Lifestyle intervention	24 weeks	Liver fat (IHTG); liver stiffness; weight/metabolic outcomes; adverse events
Khoo et al., 2017; 2019 [23,24]	Randomized pilot study	Singapore	Asian; Singapore cohort	Obese NAFLD (non-diabetic)	30	Liraglutide titrated to 3 mg daily	Diet + exercise	26 weeks intervention, 26 weeks follow-up	Liver fat (MRI); ALT/AST; CK-18/CRP; weight/metabolic outcomes; adverse events
Kuchay et al., 2020 [15]	Open-label RCT	India	Indian	T2DM + NAFLD	64	Dulaglutide 0.75 mg weekly then 1.5 mg weekly	Standard Care	24 weeks	Liver fat (MRI-PDFF); ALT/AST; liver stiffness; VTE; metabolic outcomes; adverse events
Loomba et al., 2023 [25]	Phase 2 RCT, double-blind, placebo-controlled	Multinational (Europe and USA)	Predominantly White; small Asian, Black, and Other groups reported	Biopsy-confirmed NASH cirrhosis	71	Semaglutide 2.4 mg weekly	Placebo	48 weeks	Fibrosis improvement; NASH resolution; liver fat (MRI); ALT/AST; biomarkers; adverse events
Sanyal et al., 2025 [26]	Phase 3 RCT, double-blind, placebo-controlled	Multinational (37 countries)	Mixed multinational; predominantly White and Asian, with Hispanic/Latino ethnicity reported	Biopsy-confirmed MASH (F2–F3)	1197 randomized; 800 in interim analysis	Semaglutide 2.4 mg weekly	Placebo	72 weeks	NASH resolution; fibrosis; liver fat (MRI-PDFF); ALT/AST; liver stiffness; adverse events
Wood et al., 2024 [27]	Retrospective cohort study	United States	Predominantly White	T2DM + steatotic liver disease	242	GLP-1 RA (multiple agents)	No GLP-1 RA control	3–15 months	Liver enzymes; fibrosis (FIB-4)
Zhang et al., 2020 [28]	Open-label RCT	China	Chinese	T2DM + NAFLD	60	Liraglutide 0.6 mg/day then 1.2 mg/day	Pioglitazone 15 mg/day then 30 mg/day	24 weeks	Liver fat (^1^H-MRS); ALT/AST; liver stiffness; fetuin-A; adverse events

ALT, alanine aminotransferase; AST, aspartate aminotransferase; CK-18, cytokeratin-18; CRP, C-reactive protein; FIB-4, fibrosis-4 index; GLP-1 RA, glucagon-like peptide-1 receptor agonist; IHTG, intrahepatic triglyceride content; MRI, magnetic resonance imaging; MRI-PDFF, magnetic resonance imaging–proton density fat fraction; ^1^H-MRS, proton magnetic resonance spectroscopy; NAFLD, non-alcoholic fatty liver disease; MASLD, metabolic dysfunction-associated steatotic liver disease; MASH, metabolic dysfunction-associated steatohepatitis; NASH, non-alcoholic steatohepatitis; RCT, randomized controlled trial; PSM, propensity score matching; T2DM, type 2 diabetes mellitus; VTE, venous thromboembolism.

## Data Availability

No new data was generated in the preparation of this review. All data discussed are derived from the cited published studies, which are available in the public domain through their original sources.

## Supplementary Materials

The following supporting information can be downloaded at: https://www.mdpi.com/article/10.3390/ijms27125618/s1. Reference [49] is cited in the Supplementary Materials.
