# Supplementary material for: Therapeutic Effects of Glucagon-like Peptide-1 Receptor Agonists in Non-Alcoholic Fatty Liver Disease: A Systematic Review
Source: Int J Mol Sci. 2026 Jun 22;27(12):5618. doi: 10.3390/ijms27125618 (PMC13299499; doi:10.3390/ijms27125618)
Supplement: Supplementary file 1 [file ijms-27-05618-s001.zip › ijms-4353334-supplementary.pdf]

Supplementary Material

Supplementary Table S1 - Database Search Strategies

Search String
(("Glucagon-Like Peptide 1 Receptor Agonists"[Mesh]<br>OR "GLP-1 receptor agonist*"<br>OR "glucagon-like peptide-1 receptor agonist*"<br>OR semaglutide<br>OR liraglutide<br>OR dulaglutide<br>OR exenatide<br>OR lixisenatide)<br><br>AND<br><br>("Non-alcoholic Fatty Liver Disease"[Mesh]<br>OR "Fatty Liver"[Mesh]<br>OR NAFLD<br>OR NASH<br>OR "nonalcoholic fatty liver disease"<br>OR "non-alcoholic fatty liver disease"<br>OR "nonalcoholic steatohepatitis"<br>OR "non-alcoholic steatohepatitis"))

Supplementary Table S2 - Cochrane RoB 2 Risk-of-Bias Assessment for Randomized and Interventional Studies

Study	Randomization Process	Deviations from Intended Intervention	Missing Outcome Data	Measurement of Outcome	Selection of Reported Result	Overall Risk of Bias
Newsome et al., 2021; 2024	Low	Low	Unclear	High	Low	High
Ajmera et al., 2026	Low	Low	Unclear	Low	Low	Some concerns
Armstrong et al., 2015	Low	Low	Unclear	Low	Low	Some concerns
Fan et al., 2023	Low	High	Unclear	Low	Low	High
Khoo et al., 2017; 2019	Low	Unclear	Low	Low	Unclear	Some concerns
Kuchay et al., 2020	Low	Unclear	High	Low	Low	High
Loomba et al., 2023	Low	Low	Low	Low	Unclear	Some concerns
Sanyal et al., 2025	Low	Low	Low	Low	Low	Low
L. Zhang et al., 2020	Low	HIgh	Low	High	Unclear	High

Supplementary Table S3 - Newcastle–Ottawa Scale Quality Assessment for Observational Studies

Study	Selection (0–4)	Comparability (0–2)	Outcome (0–3)	Total (0–9)	Quality
Choi et al., 2025	4	2	2--3	8--9	High
Albarmawi et al., 2025	4	2	2	8	High
Wood et al., 2024	3	1--2	2	5--6	Moderate

Supplementary Table S4 - GRADE Certainty of Evidence Assessment by Outcome

Outcome	No. of Studies	Effect Estimate	Risk of Bias	Inconsistency	Indirectness	Imprecision	Publication Bias	Certainty
Liver fat reduction	7 studies (~1,827 participants)	Consistent reduction in liver fat across MRI-PDFF / MRS studies; strongest quantitative effects in Ajmera, Kuchay, Loomba, Fan, and Zhang.	Serious	Serious	Serious	Not serious to serious	Undetected / possible	Moderate
Liver enzyme reduction (ALT/AST)	9 studies (>174,000 participants if observational included; far fewer in RCT-only synthesis)	Most studies show ALT/AST improvement, but reporting format varies widely (ratios, mean change, DiD, ETR).	Serious	Serious	Serious	Not serious	Possible	Moderate
Fibrosis improvement	5 studies (~1,642 participants if biopsy + FIB-4 studies included)	Biopsy evidence mixed: Newsome non-significant, Loomba non-significant, Armstrong shows less progression, Sanyal positive, Choi positive on FIB-4 progression.	Serious	Serious	Serious	Serious	Possible	Low
Liver stiffness	5 studies (~1,642 participants if biopsy + FIB-4	Direction generally favors GLP-1RA, but many studies are non-significant or not directly extractable;	Serious	Serious	Serious	Serious	Possible	Low

	studies included)	mixed VCTE/MRE/FibroScan endpoints.						
Resolution of steatohepatitis	4 studies (~1,640 participants)	Strongest outcome: Newsome 59% vs 17%, Armstrong 39% vs 9%, Sanyal 62.9% vs 34.3%; Loomba negative in cirrhosis.	Not serious to serious	Moderate / serious	Not serious	Not serious	Undetected / possible	Moderate to High
Hepatic inflammation and injury biomarkers	6 studies (~1,600+ participants)	Biomarker signal generally favorable: CK-18, Pro-C3, hsCRP, fetuin-A improved in several trials, but biomarkers differ substantially.	Serious	Serious	Serious	Serious	Possible	Low

Supplementary Table S5. PRISMA Checklist

Section and Topic	Item #	Checklist item	Location where item is reported
**TITLE**			
Title	1	Identify the report as a systematic review.	Title page
**ABSTRACT**			
Abstract	2	See the PRISMA 2020 for Abstracts checklist.	Abstract
**INTRODUCTION**			
Rationale	3	Describe the rationale for the review in the context of existing knowledge.	Introduction
Objectives	4	Provide an explicit statement of the objective(s) or question(s) the review addresses.	1.4
**METHODS**			
Eligibility criteria	5	Specify the inclusion and exclusion criteria for the review and how studies were grouped for the syntheses.	2.2
Information sources	6	Specify all databases, registers, websites, organisations, reference lists and other sources searched or consulted to identify studies. Specify the date when each source was last searched or consulted.	2.1, 2.2
Search strategy	7	Present the full search strategies for all databases, registers and websites, including any filters and limits used.	2.3, Supplementary Table 1
Selection process	8	Specify the methods used to decide whether a study met the inclusion criteria of the review, including how many reviewers screened each record and each report retrieved, whether they worked independently, and if applicable, details of automation tools used in the process.	2.4
Data collection process	9	Specify the methods used to collect data from reports, including how many reviewers collected data from each report, whether they worked independently, any processes for obtaining or confirming data from study investigators, and if applicable, details of automation tools used in the process.	2.5
Data items	10a	List and define all outcomes for which data were sought. Specify whether all results that were compatible with each outcome domain in each study were sought (e.g. for all measures, time points, analyses), and if not, the methods used to decide which results to collect.	2.2, Table 1, 3.1-3.7
	10b	List and define all other variables for which data were sought (e.g. participant and intervention characteristics, funding sources). Describe any assumptions made about any missing or unclear information.	2.5, Table 2
Study risk of	11	Specify the methods used to assess risk of bias in the included	2.6, 3.8,

bias assessment		studies, including details of the tool(s) used, how many reviewers assessed each study and whether they worked independently, and if applicable, details of automation tools used in the process.	Supplementary Table 2 and 3
Effect measures	12	Specify for each outcome the effect measure(s) (e.g. risk ratio, mean difference) used in the synthesis or presentation of results.	3.1-3.7
Synthesis methods	13a	Describe the processes used to decide which studies were eligible for each synthesis (e.g. tabulating the study intervention characteristics and comparing against the planned groups for each synthesis (item #5)).	2.8, Table 1
	13b	Describe any methods required to prepare the data for presentation or synthesis, such as handling of missing summary statistics, or data conversions.	2.8
	13c	Describe any methods used to tabulate or visually display results of individual studies and syntheses.	2.8, 4.7,
	13d	Describe any methods used to synthesize results and provide a rationale for the choice(s). If meta-analysis was performed, describe the model(s), method(s) to identify the presence and extent of statistical heterogeneity, and software package(s) used.	2.8
	13e	Describe any methods used to explore possible causes of heterogeneity among study results (e.g. subgroup analysis, meta-regression).	2.8, 4.7
	13f	Describe any sensitivity analyses conducted to assess robustness of the synthesized results.	NA
Reporting bias assessment	14	Describe any methods used to assess risk of bias due to missing results in a synthesis (arising from reporting biases).	2,7, supplementary table 4
Certainty assessment	15	Describe any methods used to assess certainty (or confidence) in the body of evidence for an outcome.	2.7, 3.8, supplementary table 4
**RESULTS**			
Study selection	16a	Describe the results of the search and selection process, from the number of records identified in the search to the number of studies included in the review, ideally using a flow diagram.	Section 3, figure 2
	16b	Cite studies that might appear to meet the inclusion criteria, but which were excluded, and explain why they were excluded.	Figure 2
Study characteristics	17	Cite each included study and present its characteristics.	Table 2
Risk of bias in studies	18	Present assessments of risk of bias for each included study.	3,8, supplementary 2 and 3
Results of individual studies	19	For all outcomes, present, for each study: (a) summary statistics for each group (where appropriate) and (b) an effect estimate and its precision (e.g. confidence/credible interval), ideally using structured tables or plots.	3.1 - 3.7
Results of syntheses	20a	For each synthesis, briefly summarise the characteristics and risk of bias among contributing studies.	3.1-3.8, table 2, supplementary 2 and 3

	20b	Present results of all statistical syntheses conducted. If meta-analysis was done, present for each the summary estimate and its precision (e.g. confidence/credible interval) and measures of statistical heterogeneity. If comparing groups, describe the direction of the effect.	2.8, 3.1-3.7
	20c	Present results of all investigations of possible causes of heterogeneity among study results.	4.1, 4.7
	20d	Present results of all sensitivity analyses conducted to assess the robustness of the synthesized results.	NA
Reporting biases	21	Present assessments of risk of bias due to missing results (arising from reporting biases) for each synthesis assessed.	Supplementary table 4, 3.8
Certainty of evidence	22	Present assessments of certainty (or confidence) in the body of evidence for each outcome assessed.	3.8, supplementary table 4
**DISCUSSION**			
Discussion	23a	Provide a general interpretation of the results in the context of other evidence.	4.1-4.5
	23b	Discuss any limitations of the evidence included in the review.	4.7
	23c	Discuss any limitations of the review processes used.	4.7, 4.6
	23d	Discuss implications of the results for practice, policy, and future research.	4.6, 4.8
**OTHER INFORMATION**			
Registration and protocol	24a	Provide registration information for the review, including register name and registration number, or state that the review was not registered.	2.1
	24b	Indicate where the review protocol can be accessed, or state that a protocol was not prepared.	2.1
	24c	Describe and explain any amendments to information provided at registration or in the protocol.	NA
Support	25	Describe sources of financial or non-financial support for the review, and the role of the funders or sponsors in the review.	Declarations
Competing interests	26	Declare any competing interests of review authors.	Declarations
Availability of data, code and other materials	27	Report which of the following are publicly available and where they can be found: template data collection forms; data extracted from included studies; data used for all analyses; analytic code; any other materials used in the review.	Declarations

From: Page MJ, McKenzie JE, Bossuyt PM, Boutron I, Hoffmann TC, Mulrow CD, et al. The PRISMA 2020 statement: an updated guideline for reporting systematic reviews. BMJ 2021;372:n71. doi: 10.1136/bmj.n71. This work is licensed under CC BY 4.0. To view a copy of this license, visit https://creativecommons.org/licenses/by/4.0/
